# Does Cholesterol Play a Role in the Bacterial Selectivity of Antimicrobial Peptides?

**DOI:** 10.3389/fimmu.2012.00195

**Published:** 2012-07-17

**Authors:** Jeffrey R. Brender, Austin J. McHenry, Ayyalusamy Ramamoorthy

**Affiliations:** ^1^Biophysics and Department of Chemistry, University of MichiganAnn Arbor, MI, USA

## Antimicrobial Peptides are Promising Antibiotic Compounds

The development of novel methods to overcome the inevitable resistance that develops with common antibiotics is an important area of current research. Recent studies have shown that antimicrobial peptides (AMPs) have the potential to become excellent antibiotic compounds toward a broad-spectrum of Gram-positive and Gram-negative bacteria with less potential for bacterial resistance than conventional antibiotics (Shai, 2004). Because these compounds are highly selective toward bacteria and bacteria have difficulty in developing resistance to their effects, a large number of studies have focused on designing potent AMPs for potential pharmaceutical applications (Maloy Biopolymers; Marsh et al., 2009). One of the designed peptides, MSI-78 (also known as pexiganan), rose successfully to phase II clinical trials for treating infection in the case of diabetic foot ulcer (Gottler and Ramamoorthy, 2009).

## Composition of Membranes is Key to Amp Selectivity

Bacteria have difficulty in developing resistance to AMPs because the toxicity of AMP is mostly mediated by a non-specific process rather than by an interaction with a specific protein target. Most AMPs lyse bacteria by directly interacting with the lipid bilayer of the bacterial cell membrane and disrupting the lipid bilayer structure (Oren and Shai, 1998; Epand and Vogel, 1999; Shai, 2002; Bechinger, 2011). The development of more potent and selective AMPs requires that the molecular basis of activity and selectivity be understood. Substantial progress has been made in recent years in this area, particularly using cutting-edge solid-state NMR spectroscopy to provide insights into the mechanisms of membrane disruption by AMPs (Bechinger, 1999; Durr et al., 2006; Bhattacharjya and Ramamoorthy, 2009; Ramamoorthy, 2009; Nguyen et al., 2011). For example, the high-resolution 3D structure, membrane orientation, and mechanism of membrane disruption are reported for several important peptides including LL-37 (Wildman et al., 2003; Porcelli et al., 2008), MSI-78 (Hallock et al., 2003), MSI-594 (Ramamoorthy et al., 2006; Bhunia et al., 2009), and pardaxin (Hallock et al., 2002; Porcelli et al., 2004; Bhunia et al., 2010). Biophysical studies have also revealed the role of anionic lipids, (Thennarasu et al., 2010) cholesterol, and lipopolysaccharides (Bhunia et al., 2009, 2010; Domadia et al., 2010) in Gram-negative bacteria on the antimicrobial activities of these AMPs. In addition, substantial progress has been in understanding the molecular determinants of AMP activity. For example, recent studies have shown the ability to form oligomeric aggregates in the cell membrane enhances the potency of an AMP (Toke et al., 2004; Tremouilhac et al., 2006; Marquette et al., 2008; Ramamoorthy et al., 2008; Strandberg et al., 2008). Studies have also shown that the presence of d-amino acids (Mangoni et al., 2006) and disulfide bridges (Dhople et al., 2006) can enhance resistance against proteolytic degradation without affecting the antimicrobial activity.

**Figure 1:**
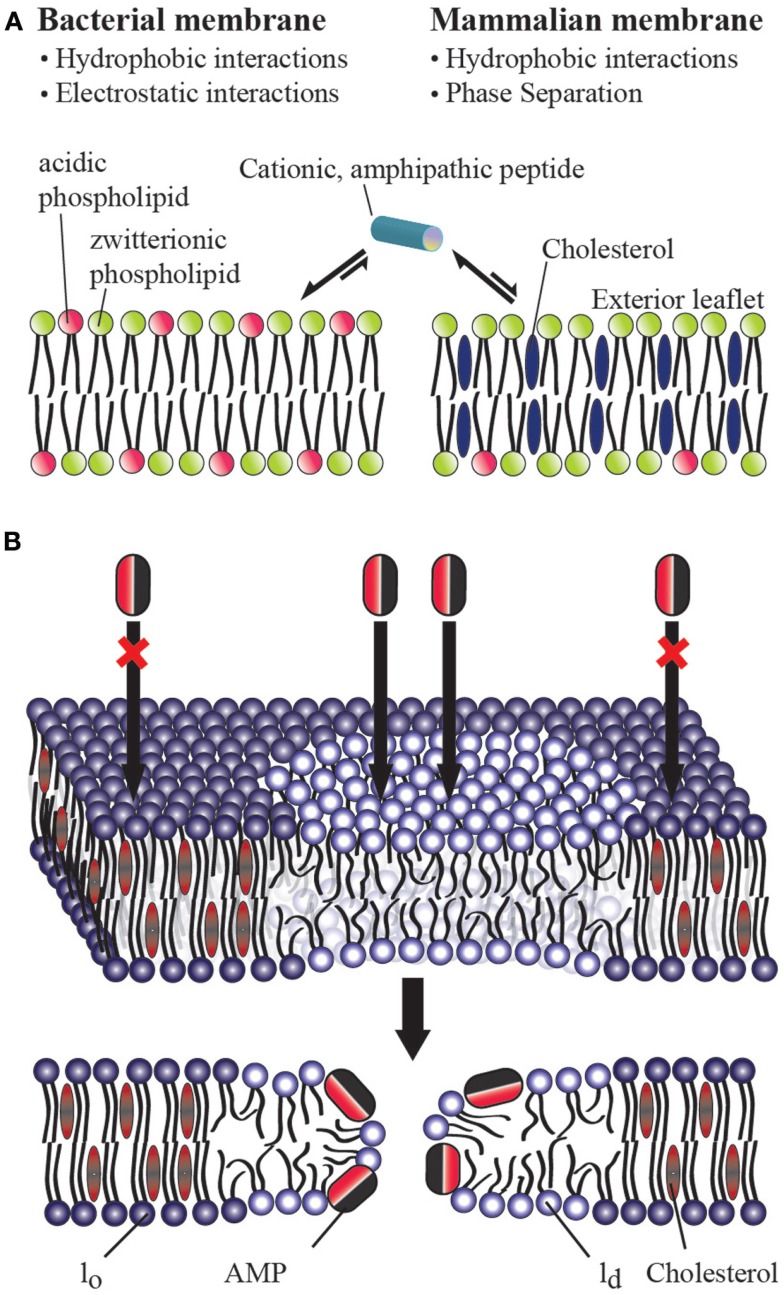
**(A)** Role of cholesterol on the bacterial selectivity of antimicrobial peptides. Lipid bilayers mimicking bacterial **(A)** and eukaryotic **(B)** cell membranes are commonly used in in vitro studies on AMPs. In eukaryotic cell membranes, the outer leaflet consists primarily of zwitterionic phosphatidylcholine lipids (such as POPC), and cholesterol (∼25%) while the inner leaflet contains anionic lipids (such as POPS). Bacterial cell membranes typically lack cholesterol and contain ∼25% acidic lipids (like POPG and cardiolipin), and ∼55% phosphatidylethanolamine (POPE). AMPs have been shown to directly interact with the lipid bilayer of bacterial cell membranes and lyse the cell by disrupting the membrane via one of the several proposed mechanisms including barrel-stave, toroidal-pore, and detergent-type disturbances. The presence of cholesterol in the eukaryotic cell membrane enhances the rigidity of lipid bilayers to inhibit the membrane disruption activities of antimicrobial peptides. The electrostatic interaction between a cationic antimicrobial peptide and the anionic lipids (POPS) present in the outer leaflet of bacterial membranes plays a vital role in bacterial selectivity and the absence of cholesterol makes the membrane disruption by an AMP easier. In the case of Gram-negative bacteria, the presence of anionic lipopolysaccharides attracts cationic AMPs. **(B)** Mechanism of action of an antimicrobial peptide in a raft-containing membrane. In a heterogeneous mixture of lipids, the presence of cholesterol in the raft domain (l_o_) resists the permeation of an antimicrobial peptide while the disordered (l_d_) lipid domain is easily disrupted by an antimicrobial peptide.

From these studies, a picture of how AMPs preferentially target bacteria has begun to emerge. The selectivity of AMPs therefore largely lies in their ability to distinguish between prokaryotic and eukaryotic membranes (Glukhov et al., 2005; Epand et al., 2006b). Biophysical studies have shown the importance of two factors in the membrane selectivity of an AMP (Figure 1A): (a) the electrostatic interaction between a cationic AMP and the acidic bacterial membrane which is composed of about ∼25% anionic lipids (POPS, POPG, and/or cardiolipin; Glukhov et al., 2005; van Meer et al., 2008; Epand et al., 2010) and (b) the presence of a large amount of cholesterol in a eukaryotic cell membrane which inhibits membrane disruption by rigidifying the lipid bilayer structure (Benachir et al., 1997; Matsuzaki, 1999; Glukhov et al., 2005; Epand et al., 2006a; Verly et al., 2008). These factors controlling the membrane selectivity of AMPs can also be exploited for other pharmaceutical targets. For example, several AMPs have been shown to have anticancer activities; this property has been attributed to the presence of anionic lipids in the outer leaflet of the cancer cell plasma membrane (Hoskin and Ramamoorthy, 2008). Similarly, most AMPs also kill fungi, protozoa, and even enveloped viruses, which all show a lipid distribution different than a normal eukaryotic cell (Oren and Shai, 1998; Epand and Vogel, 1999; Shai, 2002; Bechinger, 2011; Nguyen et al., 2011; Pius et al., 2012). Despite this progress in understanding the molecular determinants of AMP activity, there are still unresolved questions, particularly with regards to the preferential targeting of bacterial membranes. While the role of anionic lipids in membrane targeting of AMPs is well established, the role of cholesterol is still not clear. Accordingly, this opinion article focuses on the distinct roles of cholesterol in homogenous versus heterogeneous lipid bilayers.

## Cholesterol is Believed to Play a Role in Bacterial Selectivity of AMPs

One of the major differences between bacterial and eukaryotic cell membranes is the presence of a large amount of cholesterol in eukaryotic cell membranes and a complete absence in bacterial cell membranes (Figure 1A). Cholesterol has been shown to protect human erythrocytes from attack by magainin 2 (Matsuzaki et al., 1995b). Similar studies on model membranes have demonstrated that the presence of cholesterol reduces AMP binding and suppresses the disruption of lipid bilayer structure by AMPS (Feigin et al., 1995; Matsuzaki et al., 1995a; Tytler et al., 1995; Raghuraman and Chattopadhyay, 2004; Glukhov et al., 2005; Verly et al., 2008; Wu et al., 2010). Solid-state NMR studies have provided high-resolution insights into the role of cholesterol against the function of several AMPs (Benachir et al., 1997; Wildman et al., 2003; Ramamoorthy et al., 2010). Cholesterol is known to increase membrane cohesion and mechanical stiffness (Evans and Waugh, 1977; Henriksen et al., 2006) which may resist the membrane bending required for many AMPs to function (Allende et al., 2005). This interaction reduces the tilt of the paradaxin helix relative to the bilayer normal, which in turn reduces the stability of the paradaxin pore (Ramamoorthy et al., 2010). However, for most AMPs a noticeable inhibitory effect of cholesterol is only noticeable after the formation of liquid ordered lipid phase at high concentrations of cholesterol (∼20%; McHenry et al., submitted) which suggests it may be due to an indirect effect due to a modulation of membrane properties rather than a direct interaction (Feigin et al., 1995). Despite these advances, the actual reason for the reduced affinity of many AMPs for cholesterol containing membranes is not fully understood. As noted above, this is traditionally been interpreted as a consequence of the increased acyl chain order in the liquid ordered phase of cholesterol containing membranes. In this context, it is interesting to compare cholesterol's effects on AMPs which do not clearly prefer the disordered liquid crystalline lipid phase or ordered gel phase. Surprisingly, cholesterol still strongly inhibits these peptides, which suggests an additional factor, such as dehydration of the headgroup region (M’Baye et al., 2008) is partially responsible for cholesterol's effect.

## Cholesterol Loses Its Effectiveness in Inhibiting AMPs When Incorporated into Raft-Like Domains

While biophysical studies have shown the ability of cholesterol to suppress the action of an AMP against a homogeneous lipid bilayer, recent studies have revealed that cholesterol does not have this same effect in heterogeneous lipid systems (Pokorny and Almeida, 2005; Pokorny et al., 2006). Though few studies have looked at membrane disruption by AMPs in heterogeneous systems with phase separation [particularly in liquid ordered (*l*_o_) liquid-disordered (*l*_d_) domain coexistences often referred to as “lipid rafts”], two studies by the Almeida group demonstrated the permeabilizing activity of δ-lysin in raft-like palmitoyl-2-oleoylphosphatidylcholine/cholesterol/sphingomyelin (POPC/Chol/SM) mixtures (Pokorny and Almeida, 2005; Pokorny et al., 2006). These studies revealed that membrane permeabilization by δ-lysin occurs exclusively in the *l*_d_ phase in membranes with *l*_d_ − *l*_o_ phase segregation and that the localization of δ-lysin to the *l*_d_ phase results in greater membrane disruption than would be expected in the absence of phase segregation. Our own group recently demonstrated that this important effect occurs among a diverse set of AMPs (MSI-78, MSI-594, MSI-843, and MSI-367) encompassing several membrane disruptive mechanisms (McHenry et al., submitted). These combined results indicate that the phase separation naturally occurring in eukaryotic membranes is likely to nullify the effect of cholesterol against membrane disruption by AMPs. This surprising result suggests either cholesterol is not as important in determining the selectivity of AMPs toward bacterial membranes as once supposed, or unknown additional factors mitigate this effect in eukaryotic cells.

The mechanism of action of an AMP in a heterogeneous lipid system is depicted in Figure 1B. These results suggest that raft formation localizes the concentration of cholesterol in the cell membrane in such a way that non-raft domains of the cell membrane can be easily disrupted by AMPs and toxins. It is also possible that the phase behavior of the membrane and the physicochemical properties of the boundaries connecting the ordered and disordered domains play important roles in the membrane disruption process by AMPs. For instance, paradaxin has been shown to segregate a homogeneous membrane into cholesterol rich and cholesterol poor domains (Epand et al., 2006a). While the AMPs that have been investigated so far function by the non-specifically mechanically disrupting the membranes (carpet, detergent-type, or toroidal-pore formation) mechanism (Figure 1A), it is unclear how AMPs resembling more traditional ion channels (barrel-stave mechanism) would interact with heterogeneous lipid systems (Figure 1B). Therefore, it is important to further investigate the interaction of a variety of AMPs with more heterogeneous lipid systems.

## Future Scope

While the development of AMPs for antibiotic applications is highly important, it is essential to understand the origin of their bacterial selectivity. As mentioned above, recent studies have shown that AMPs easily disrupt the structure of heterogeneous lipid systems, and therefore cholesterol is unlikely to play a major role in reducing the toxicity or increasing the selectivity of AMPs. Since a natural eukaryotic cell membrane contains heterogeneous lipid systems and domains, cholesterol poor domains must be easily disruptable by an AMP. Further studies probing the role of cholesterol in different types of lipid bilayers with a variety of AMPs are essential to better understand the exact role of cholesterol on the toxicity and selectivity of AMPs. Such studies would aid in the design of more efficient AMPs.
